# Alternation of Sound Location Induces Visual Motion Perception of a Static Object

**DOI:** 10.1371/journal.pone.0008188

**Published:** 2009-12-07

**Authors:** Souta Hidaka, Yuko Manaka, Wataru Teramoto, Yoichi Sugita, Ryota Miyauchi, Jiro Gyoba, Yôiti Suzuki, Yukio Iwaya

**Affiliations:** 1 Department of Psychology, Graduate School of Arts and Letters, Tohoku University, Sendai, Miyagi, Japan; 2 Core Research for Evolutional Science and Technology (CREST), Japan Science and Technology Agency, Tokyo, Japan; 3 Research Institute of Electrical Communication, Tohoku University, Sendai, Miyagi, Japan; 4 Neuroscience Research Institute, National Institute of Advanced Industrial Science and Technology (AIST), Tsukuba, Ibaraki, Japan; University of Sydney, Australia

## Abstract

**Background:**

Audition provides important cues with regard to stimulus motion although vision may provide the most salient information. It has been reported that a sound of fixed intensity tends to be judged as decreasing in intensity after adaptation to looming visual stimuli or as increasing in intensity after adaptation to receding visual stimuli. This audiovisual interaction in motion aftereffects indicates that there are multimodal contributions to motion perception at early levels of sensory processing. However, there has been no report that sounds can induce the perception of visual motion.

**Methodology/Principal Findings:**

A visual stimulus blinking at a fixed location was perceived to be moving laterally when the flash onset was synchronized to an alternating left-right sound source. This illusory visual motion was strengthened with an increasing retinal eccentricity (2.5 deg to 20 deg) and occurred more frequently when the onsets of the audio and visual stimuli were synchronized.

**Conclusions/Significance:**

We clearly demonstrated that the alternation of sound location induces illusory visual motion when vision cannot provide accurate spatial information. The present findings strongly suggest that the neural representations of auditory and visual motion processing can bias each other, which yields the best estimates of external events in a complementary manner.

## Introduction

In order to establish coherent and robust percepts of our surroundings, the perceptual system appropriately and flexibly combines or integrates multisensory inputs [1], depending on the accuracy and/or reliability of each input [2]. The adaptation to a looming visual stimulus induced not only the motion aftereffect (MAE) in visual modality but also the aftereffect in auditory modality, that is, a sound of fixed intensity was perceived as decreasing in intensity [3]. When an auditory moving stimulus was presented in conjunction with visual motion, the auditory stimulus was perceived to move in the same direction as the visual moving stimulus, even if the auditory stimulus actually traveled in the opposite direction [4]. These studies suggest that there are multimodal contributions to motion perception.

It has been reported that auditory information affects visual motion perception. When two visual stimuli moved across each other, a streaming or bouncing perception was equally likely to occur. In this stream-bounce display, a transient auditory stimulus dominantly induced a bouncing perception [5], [6]. Similarly, in an ambiguous visual motion display where left or right motion perception occurred equally often, a transient auditory signal captured the temporal positional information of a visual stimulus so that the visual stimulus appeared to move in a certain direction [7]. These studies revealed the modulatory, but not driving or inducing, effects of auditory information on visual motion perception. Here, we demonstrate that auditory signals can induce motion perception of a static visual stimulus. A visual stimulus blinking at a fixed location was perceived to be moving laterally when its flash onset was synchronized to an alternating left-right sound source. We found that this illusory motion could be observed more frequently in peripheral vision where spatial resolutions are lower (see Supplemental Video S1 for a demonstration). We call this phenomenon “Sound-Induced Visual Motion (SIVM),” hereafter.

## Results

### Effects of Eccentricity

**Figure 1:**
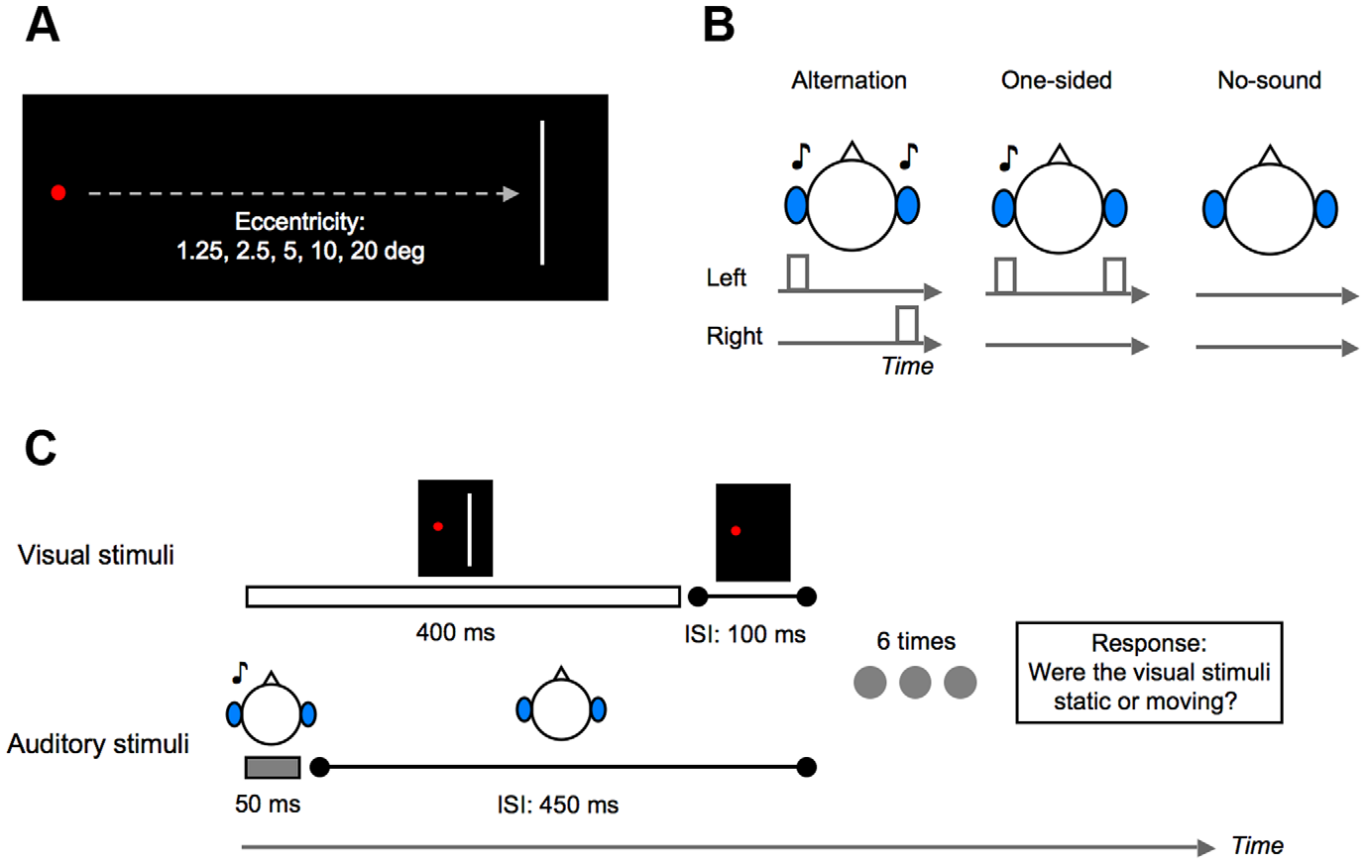
Visual and auditory stimuli used in Experiment 1. (A) Visual stimuli (white bars) were presented with the various eccentricities from a fixation point. (B) Auditory stimuli (white noise bursts) were presented as alternating or to be fixed at one side of both the ears, and no sound was also presented. (C) A visual stimulus was presented six times, synchronized with an auditory stimulus. The participants' task was to report whether or not they perceived visual motion.

First, we investigated the spatial aspects of the SIVM (Experiment 1). A visual stimulus (a white bar, with 400 ms duration) was presented six times with 500 ms of stimulus onset asynchrony (SOA), synchronized with an auditory stimulus (a white noise burst, with 50 ms duration) in each trial. The onset of the sound coincided with the presentation of the visual stimuli. The retinal eccentricity of the visual stimuli was varied randomly from 1.25 deg to 20 deg per trial (Figure 1A). There were three conditions for sound presentation. In the alternate condition, the auditory stimuli were presented alternately to the left and right ears via headphones. In the one-sided condition, we presented the auditory stimuli to either the left or the right ear to examine the effect of the presentation of the sound itself. Finally, no sound was presented in the no-sound condition (Figure 1B). Participants were asked to report whether or not they perceived visual motion (Figure 1C).

**Figure 2:**
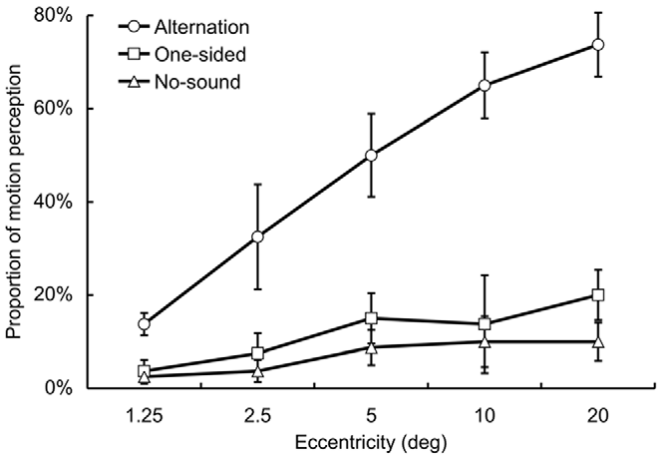
Results of Experiment 1 with the manipulation of the eccentricities of visual stimuli (N = 4). Error bar denotes the standard error of the mean.

The blinking bar appeared to be moving laterally when presented in conjunction with the sounds alternating on the left and right side. This tendency was gradually strengthened with an increasing retinal eccentricity. However, this illusory visual motion was not observed when the sounds were presented only from one side or without sounds, indicating that the alternation of sound location between the ears is important for the SIVM (Figure 2). We conducted a repeated analysis of variance (ANOVA) with two within-participant factors: eccentricities (1.25, 2.5, 5, 10, and 20 deg) and three auditory conditions (alternation, one-sided, and no-sound). The ANOVA revealed a significant main effect of eccentricities (*F*_4, 12_ = 7.96, *p*<.005). In addition, a main effect of auditory conditions was significant (*F*_2, 6_ = 37.09, *p*<.001). An interaction effect between these factors was also significant (*F*_8, 24_ = 9.21, *p*<.001). A post-hoc test (Tukey's HSD, *p*<.05) revealed that the proportion of motion perception was higher in the alternation condition than the other conditions for all the eccentricities, except for 1.25 deg. These results indicate that the alternation of sound location induces the visual motion perception of the static visual stimulus, and the effect of auditory signals became greater with the increment of the eccentricity of the visual stimuli.

### Quantification of the SIVM

We further quantified the strength of the SIVM using a motion nulling procedure with the method of constant stimuli (Experiment 2). Our preliminary experiment revealed that individual performances were highly consistent at 10 deg. Thus, the eccentricity of the visual stimuli was fixed at 10 deg in this experiment and also in a following experiment. Two visual stimuli (with 400 ms duration in each) were presented with 500 ms of SOA between them, synchronized with two auditory stimuli (with 50 ms duration in each). The auditory stimuli were alternated from the left to the right ears (rightward sound condition) or the right to the left ears (leftward sound condition). The no-sound condition was also included. The visual stimulus was displaced 0.05, 0.1, 0.2, or 0.4 deg from left to right or vice versa. The participants were asked to report the perceived motion direction of the visual stimuli (left or right).

**Figure 3:**
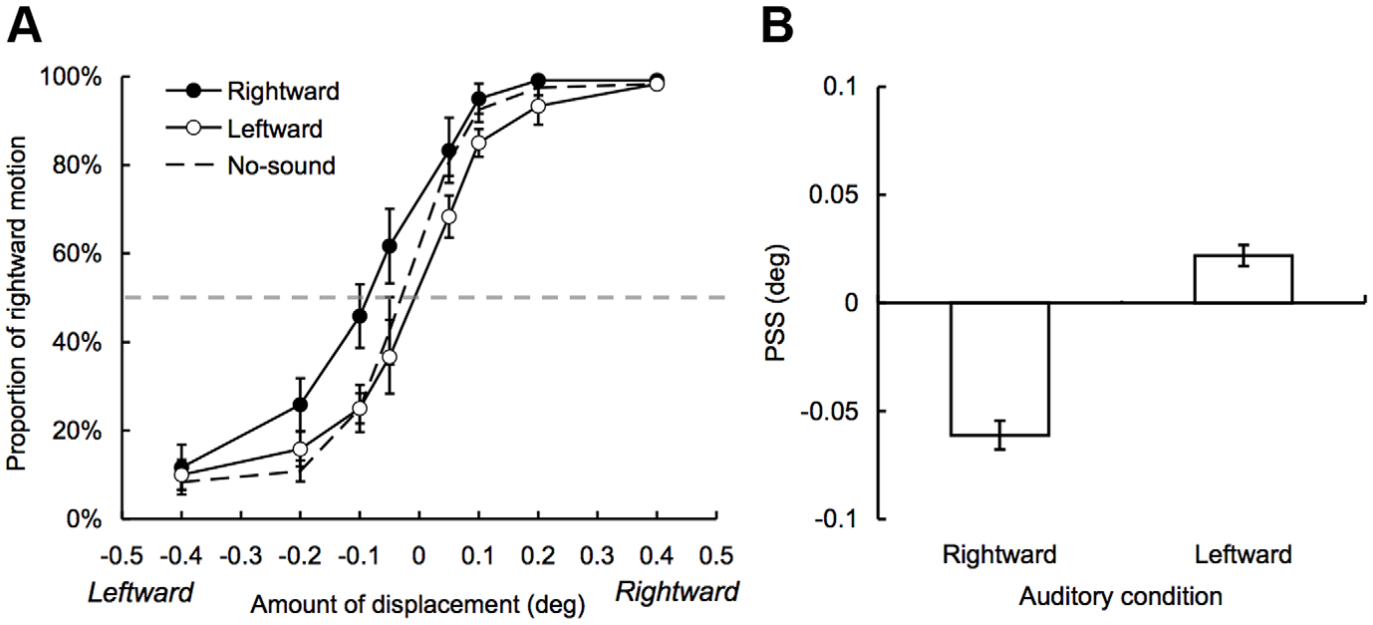
Results of Experiment 2 (N = 6). (A) The proportion of rightward motion perception of visual stimuli as a function of the amount of physical displacements of visual stimuli. Whereas negative values indicate leftward visual motion, positive values indicate rightward visual motion in the horizontal axis. Each psychometric function represents each sound condition. A gray dashed line indicates 50% threshold. (B) The normalized points of subjective stationarity (PSS) against that for the no-sound condition. Error bar denotes the standard error of the mean.

We calculated a point of subjective stationarity (PSS) where the visual stimulus was perceived to be static (i.e., 50% threshold of psychometric functions, see Figure 3A) by fitting a cumulative Gaussian distribution function to each participant's data using a maximum likelihood method. Figure 3B shows the PSSs for the leftward and rightward sound conditions normalized against that for the no-sound condition. The PSS shifted in the direction of the leftward visual motion for the rightward sound condition and the rightward visual motion for the leftward sound condition. A repeated ANOVA with a within-participant factor revealed that the main effect of auditory conditions was significant (*F*_2, 10_ = 17.28, *p*<.001). A post-hoc test (Tukey's HSD, *p*<.05) revealed significant differences in PSS between the rightward and leftward sound conditions and between the rightward sound and no-sound conditions. These results indicate that the alternation of sound location induces the motion perception of the visual stimuli toward the alternate direction in the SIVM.

### Effects of Stimulus Onset Asynchrony

In the third experiment (Experiment 3), we investigated the temporal aspects of the SIVM. The eccentricity of the static visual stimuli was fixed at 10 deg. The auditory stimuli were always presented alternately to the left and right ears. Two visual (with 400 ms duration in each) and two auditory (with 50 ms duration in each) stimuli were presented. The SOA between the two visual (and auditory) stimuli was 500 ms and that between the visual and auditory stimuli was varied randomly from −250 ms (auditory stimulus first) to +250 ms (visual stimulus first) in 50 ms steps. The participants were asked to report the perceived motion direction of the visual stimuli (left or right).

**Figure 4:**
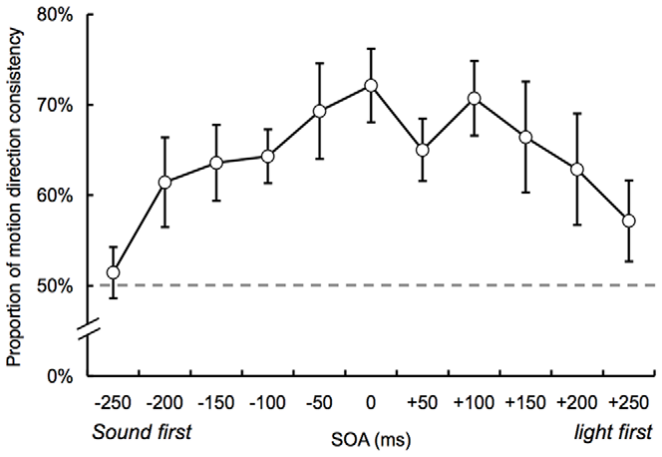
Results of Experiment 3 with the manipulation of SOA (N = 7). The vertical axis denotes the proportion that the perceived motion direction of the visual stimuli was consistent with the alternate direction of the sounds between the ears. While negative SOAs indicate that the auditory stimuli were presented earlier than the visual stimuli, the visual stimuli were presented earlier than the auditory stimuli in positive SOAs. Error bar denotes the standard error of the mean.

Figure 4 shows the proportion of trials that the perceived motion direction of the visual stimuli was consistent with the alternate direction of the sounds between the ears. It appears from the figure that the proportion of the direction consistency gradually decreases with the increment of SOA around 200 ms. We conducted a repeated ANOVA with a within-participants factor (SOA). This revealed a significant main effect (*F*_10, 60_ = 2.45, *p*<.05). Moreover, it seems that the proportion of the direction consistency peaks around 0 ms of SOA. We estimated the peak values of the SOA functions by fitting a quadratic function to each participant's data using a least square method. The average peak is 24.86 ms (corresponds to visual stimulus first) and the 95% confidence interval is from −32.55 ms to 82.27 ms, which was calculated by multiplying the standard error across observers by 1.96. These results indicate that the alternation of sound location can induce the consistent visual motion for the static stimulus within a certain temporal range.

## Discussion

In the present study, we demonstrated that a static visual stimulus blinking in one place was perceived to be moving laterally when it was accompanied with the alternation of sound location in the left and right ears. The SIVM was strengthened with an increasing retinal eccentricity (2.5 deg to 20 deg), and frequently occurred when the onsets of visual and auditory stimuli were synchronized. We also found that the alternation of sound location induced the motion perception of the static visual stimuli toward the alternate direction of the sounds. These results confirm that the alternation of sound location can induce the visual motion perception of static visual stimuli in the case where the spatial resolution of visual information is low and the temporal consistency between the audiovisual stimuli is maintained.

It could be assumed that the effect of eye movements might be involved in the SIVM. In our pilot experiments, we found that eye movements themselves could induce illusory visual motion perception of the static visual stimuli depending on the participants' ability of firm fixation. To avoid this confounding effect, we introduced training sessions before the experimental sessions, in which the participants were asked to firmly stare at a fixation point and were trained to discriminate between static and moving visual stimuli without hearing the sounds (see Materials and Methods section). It might be also considered that the alternation of sound location in the left and right ears would induce eye movements. In order to test this possibility, we recorded eye movements and discarded the trials in which eye position deviated by more than 1 deg of the visual angle from the center of a fixation point. We confirmed that the SIVM reliably occurred without eye movements (see Supplemental Figure S1). It can be therefore concluded that eye movements are not a decisive factor for the current findings.

A previous investigation showed that an auditory spatial cue could capture the spatial attention in the visual modality [8]. It was also demonstrated that auditory spatial cues induce the motion perception of a visual stimulus away from the cued location [9]. One would conjecture that these attentional cueing effects in space might be related to the SIVM demonstrated in the present study. It is plausible that an auditory attentional cue certainly induces the illusory visual motion since the visual percept for the stimulus might gradually develop from the cued side [9]. However, the perceptual quality of motion in the SIVM is quite different from that in the attentional cueing effect: the movement of the static visual stimuli appears in synchronization with the alternation of sound location with no delay in the SIVM (see Supplemental Video S1 for the demonstration). The difference is also observed in temporal aspects. It has been reported that the auditory spatial cues can be effective only when they preceded 100 ms to 300 ms [8] or 0 ms to 300 ms [9] to a visual stimulus. This temporal aspect is inconsistent with the present results (Experiment 3), in which the SIVM also occurred frequently at positive SOAs (i.e., the visual stimuli preceded the auditory ones). Thus, the involvement of attentional mechanisms can hardly explain all of the present findings.

It could also be suspected that the SIVM might result from some response biases. For example, the participants might expect that they perceive visual stimuli to move whenever the alternation of sound location between the ears occurs. If this were the case, the motion direction judgments for visual stimuli should have always been consistent with the alternate direction of the sounds between the ears. However, the proportion of consistent judgments of motion direction varied with SOAs between the visual and auditory stimuli in Experiment 3, suggesting that the occurrence of the SIVM would depend on the temporal consistency between the visual and auditory stimuli. Moreover, an additional experiment showed that the occurrence of the SIVM differed depending on the inter-stimulus intervals between the audiovisual stimuli, and that the effect was not identical among the participants (see Supplemental Figure S2). These results suggest that the SIVM was affected by the temporal distance between the audiovisual stimuli, and that the effectiveness of cues contained in the alternation of sound location (motion, displacement, and so on) would be individually different. Therefore, the SIVM cannot be explained by the response biases alone.

We assume that the SIVM occurs by audiovisual interactions. It has been suggested that multimodal inputs interact depending on the accuracy and/or reliability of each input [2]. Consistent with this idea, the alternation of sound location can induce the illusory visual motion more strongly at large retinal eccentricities at which visibility or visual sensitivity degrades. We could assume that motion/displacement cues contained in the alternation of sound location can be involved in the SIVM. With regard to motion cue, we could consider that the auditory apparent motion signals induced by the alternation of sound location between the ears directly induce the visual motion perception (cf. *visual* motion capture, [10], [11]). A previous study indicates that motion signals are more salient than static signals [12]. This suggests that the auditory apparent motion signal can override the perception of static visual stimuli and directly trigger or drive the visual motion processing, depending on the reliability or the saliency of visual information. On the other hand, it could be also considered that the displacement cue of sounds delivered from the left and right ears can successively induce the mislocalization of static visual stimuli with vulnerable positional information in peripheral vision so that motion perception occurs. Further research would clarify the mechanisms involved in the SIVM.

In summary, the present study clearly demonstrates that the alternation of sound location can induce visual motion (SIVM), especially at large retinal eccentricities. We confirmed that the SIVM is unattributable to eye movements, response biases, or attentional modulations. Rather, the SIVM is assumed to be a product of the audiovisual interaction in which the motion/displacement cues contained in the alternation of sound location trigger motion perception to peripheral visual inputs with vulnerable positional information.

## Materials and Methods

### Ethics Statement

Written consent was obtained from each participant prior to the experiments. The experiments were approved by the local ethics committee of Tohoku University.

### Participants and Apparatus

There were four, six, and seven volunteers, all of which included two of the authors (S.H. and W.T.), who participated in Experiments 1, 2, and 3, respectively. All participants had normal or corrected-to-normal vision and normal hearing. The visual stimuli were presented on a CRT display (Sony Trinitron GDM-FW900, 24 inch) with a resolution of 1600×1200 pixels and a refresh rate of 75 Hz. The auditory stimuli were presented through an audio interface (Rolland EDIROL FA-66) and headphones (SENNHEISER HDA200). A customized PC (Dell-Dimension 8250) and MATLAB (The Mathworks, Inc.) with the Psychophysics Toolbox [13], [14] were used to control the experiment. We confirmed that the onset of the visual and auditory stimuli was synchronized using a digital oscilloscope (IWATSU TS-80600). The participants were instructed to place their heads on a chin rest. All the experiments were conducted in a dark room.

### Stimuli

A red circle (0.4 deg in diameter; 17.47 cd/m^2^) was presented as a fixation point on a black background. A sequence of white bars (3 deg ×0.2 deg; 4.99 cd/m^2^) was presented as visual stimuli at an eccentricity of either 1.25, 2.5, 5, 10, or 20 deg in Experiment 1 and 10 deg in Experiments 2 and 3 in the participants' dominant eye field. Each white bar was presented for 400 ms and the inter-stimulus interval (ISI) of the white bars was 100 ms (Figure 1). A white noise burst was presented as an auditory stimulus for 50 ms with a cosine ramp of 5 ms at the onset and offset (sound pressure level: 85 dB, sampling frequency: 22050 Hz). The white noise bursts were created per trial. In Experiment 1, six white noise bursts were sequentially presented alternately to the left and right ears in the alternate condition and to either the left or the right ears in the one-sided condition. In Experiments 2 and 3, only two white noise bursts were presented alternately to the left and right ears in the alternate condition. The situation without any sound was also tested (the no-sound condition) in Experiments 1 and 2. The onset timing of each white noise burst was always synchronized with that of the visual stimulus (white bar) regardless of the conditions in Experiments 1 and 2. In Experiment 3, the SOA of each white noise burst from each white bar was varied from –250 ms to 250 ms in 50 ms steps. While negative SOAs indicate that the auditory stimuli were presented earlier than the visual stimuli, the visual stimuli were presented earlier than the auditory stimuli in positive SOAs.

### Procedure

After the presentation of the fixation point for 500 ms, the visual and auditory stimuli were presented six times in Experiment 1 and two times in Experiments 2 and 3. The participants' task was to report whether they perceived visual motion in Experiment 1 and to report the perceived motion direction of visual stimuli (left or right) in Experiments 2 and 3.

All of the experiments included a training session and the main experimental sessions. In the training session of Experiment 1, the participants were asked to discriminate between static and moving visual stimuli for 100 trials: Visual stimuli (2; static/moving) × Eccentricities (5) × Repetitions (10). The white bar was displaced back and forth by 0.2 deg in the horizontal direction when it moved. The training session was repeated until the discrimination performance reached above 75% for each eccentricity. In the training session of Experiments 2 and 3, the participants performed the same discrimination task as in the training session of Experiment 1 for 20 trials: Visual stimuli (2; static/moving) × Repetitions (10). The eccentricity of the visual stimuli was always at 10 deg.

The main session was separated into two in each experiment. In Experiment 1, the main session consisted of 300 trials where visual stimuli were always static: Eccentricities (5) × Auditory stimuli (3) × Repetitions (20). In the alternate condition, the first sound was delivered to the right ear for the half of the trials and to the left ear for the other half. In the one-sided condition, sounds were delivered to the right ear for the half of the trials and to the left ear for the other half. The presentation order of the conditions was randomized in the main session. Additionally, 120 filler trials where the white bar was actually displaced by 0.2 deg in the horizontal direction were randomly introduced in the trials of the main session: Eccentricities (5) × Auditory stimuli (3) × Repetitions (8). In the filler trials, the initial onset position (left or right) of the white bar was consistent with those of the sound in the alternate and one-sided conditions, and randomly assigned in the no-sound condition.

In Experiment 2, the main session consisted of 480 trials: Auditory stimuli (3; leftward/rightward/no-sound) × Amount of displacement of the visual stimuli (4; 0.05, 0.1, 0.2, and 0.4 deg) × Direction consistency between the alternation of sound location and the visual motion (2) × Repetitions (20). The first sound was delivered to the right ear for the half of the trials and to the left ear for the other half. The presentation order of the conditions was randomized.

In Experiment 3, the main session included 220 trials where visual stimuli were always static: SOAs (11; from −250 ms to +250 ms in 50 ms steps) × Repetitions (20). The first sound was delivered to the right ear for the half of the trials and to the left ear for the other half. The presentation order of the conditions was randomized in the main session. We pooled and analyzed 20 sets of data in each SOA in the main session. Additionally, 88 trials were randomly introduced as fillers where visual stimuli actually moved in the horizontal direction: SOAs (11) × Direction consistencies between the alternation of sound location and the visual motion (2; consistent/inconsistent) × Repetitions (4). In these filler trials, a displacement direction of the white bar was randomly assigned and counterbalanced among the conditions.

## Supporting Information

Figure S1 (A) Proportion of visual motion perception except for trials in which eye position deviated by more than 1 deg from the center of a fixation (N = 3). The static visual stimuli were presented six times at 10 deg of retinal eccentricity in conjunction with the alternating sounds between the left and right ears (sound condition) or without any sound (no-sound condition). We monitored the positions of the right eye at a sampling rate of 30 Hz using a CCD camera, recording the reflectance of infrared LED lights from pupil. We discarded 15+/−7 (SEM) % and 21+/−9 (SEM) % of trials in the sound and no-sound conditions, respectively. SIVM reliably occurred without eye movements. In addition, we confirmed that the correlation between the amplitudes of eye movements (root-mean-square average over time) and motion perception was not positive in the sound condition (r = −0.22+/−0.11 (SEM)). (B) Examples of eye movement recording data (10 trials) for a participant in the sound condition. Each colored line shows the time course of eye positions in the horizontal direction when the SIVM occurred (left) and did not occur (right) without the eye movements more than 1 deg from the center of a fixation.
(2.44 MB TIF)

Figure S2 Typical data (two participants) obtained from an experiment by manipulating the inter-stimulus intervals (ISIs) between audio-visual stimuli. The eccentricity of the static visual stimuli was fixed at 10 deg. A visual stimulus was presented six times, synchronized with an auditory stimulus. The duration of both stimuli was fixed at 50 ms. The ISIs of the auditory and visual stimuli were varied randomly from 50 ms to 400 ms with 50 ms steps. The participants were asked to report whether they perceived motion with the visual stimuli. As shown in the figures, we found that the effect of ISIs differed for each participant: the SIVM occurred mainly in shorter ISIs for some participants (left), whereas in longer ISIs for other participants (right). These results suggest that the temporal distance between the audiovisual stimuli affects the occurrence of the SIVM.
(1.30 MB TIF)

Video S1 The demonstration of the SIVM. The sounds were desirable to be provided through headphones or earphones. Please fixate on the red circle when it appears. A white bar repeatedly appears at a fixed position without sounds in the first sequence. In the following sequences, the sounds alternate between the left and right ears. Then, a white bar appears to move laterally although it is actually stationary at a fixed position. In some sequences, the white bar actually moves. However, it may be difficult to distinguish this from the SIVM.
(2.72 MB MOV)
