# Supplementary figures and images for: Alternation of Sound Location Induces Visual Motion Perception of a Static Object
Source: PLoS One. 2009 Dec 7;4(12):e8188. doi: 10.1371/journal.pone.0008188 (PMC2781159; doi:10.1371/journal.pone.0008188)

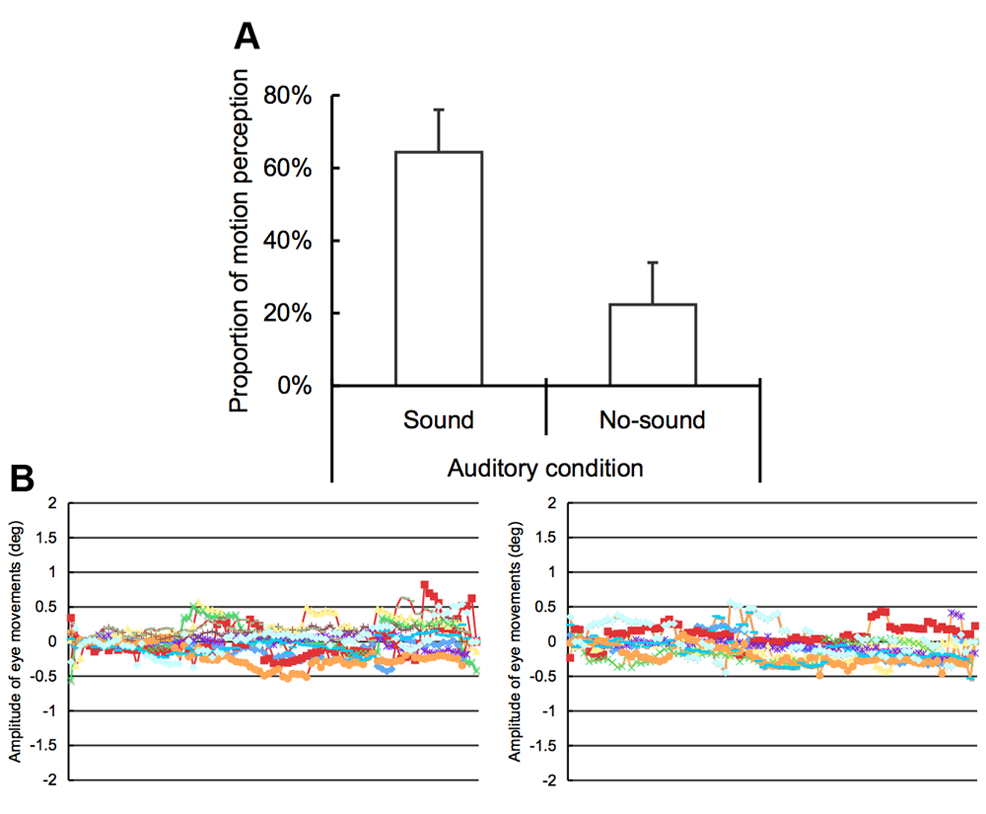

Supplement: Figure S1 — (A) Proportion of visual motion perception except for trials in which eye position deviated by more than 1 deg from the center of a fixation (N = 3). The static visual stimuli were presented six times at 10 deg of retinal eccentricity in conjunction with the alternating sounds between the left and right ears (sound condition) or without any sound (no-sound condition). We monitored the positions of the right eye at a sampling rate of 30 Hz using a CCD camera, recording the reflectance of infrared LED lights from pupil. We discarded 15+/−7 (SEM) % and 21+/−9 (SEM) % of trials in the sound and no-sound conditions, respectively. SIVM reliably occurred without eye movements. In addition, we confirmed that the correlation between the amplitudes of eye movements (root-mean-square average over time) and motion perception was not positive in the sound condition (r = −0.22+/−0.11 (SEM)). (B) Examples of eye movement recording data (10 trials) for a participant in the sound condition. Each colored line shows the time course of eye positions in the horizontal direction when the SIVM occurred (left) and did not occur (right) without the eye movements more than 1 deg from the center of a fixation. (2.44 MB TIF) [file pone.0008188.s001.tif]

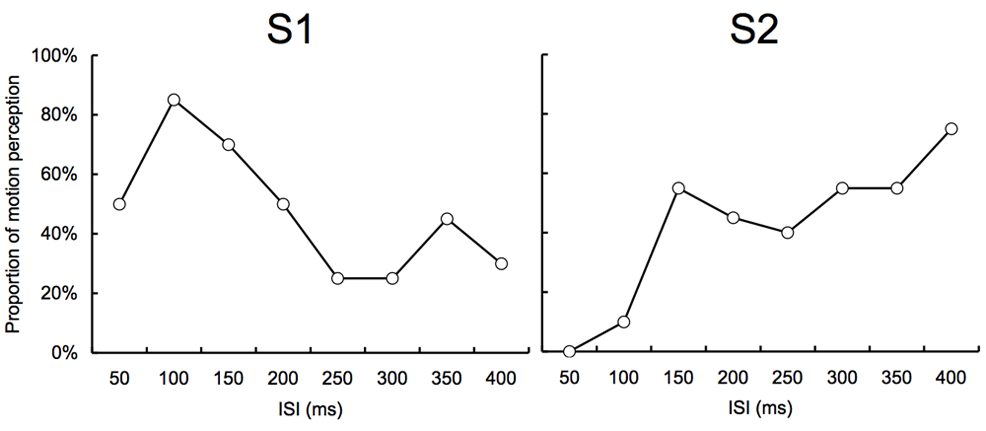

Supplement: Figure S2 — Typical data (two participants) obtained from an experiment by manipulating the inter-stimulus intervals (ISIs) between audio-visual stimuli. The eccentricity of the static visual stimuli was fixed at 10 deg. A visual stimulus was presented six times, synchronized with an auditory stimulus. The duration of both stimuli was fixed at 50 ms. The ISIs of the auditory and visual stimuli were varied randomly from 50 ms to 400 ms with 50 ms steps. The participants were asked to report whether they perceived motion with the visual stimuli. As shown in the figures, we found that the effect of ISIs differed for each participant: the SIVM occurred mainly in shorter ISIs for some participants (left), whereas in longer ISIs for other participants (right). These results suggest that the temporal distance between the audiovisual stimuli affects the occurrence of the SIVM. (1.30 MB TIF) [file pone.0008188.s002.tif]
